# Estimating the Active Space of Male Koala Bellows: Propagation of Cues to Size and Identity in a *Eucalyptus* Forest

**DOI:** 10.1371/journal.pone.0045420

**Published:** 2012-09-20

**Authors:** Benjamin D. Charlton, David Reby, William A. H. Ellis, Jacqui Brumm, W. Tecumseh Fitch

**Affiliations:** 1 Department of Cognitive Biology, University of Vienna, Vienna, Austria; 2 School of Psychology, University of Sussex, Brighton, East Sussex, United Kingdom; 3 Koala Research Centre of Central Queensland, Central Queensland University, Rockhampton, Queensland, Australia; 4 Lone Pine Koala Sanctuary, Brisbane, Queensland, Australia; University of Bristol, United Kingdom

## Abstract

Examining how increasing distance affects the information content of vocal signals is fundamental for determining the active space of a given species’ vocal communication system. In the current study we played back male koala bellows in a *Eucalyptus* forest to determine the extent that individual classification of male koala bellows becomes less accurate over distance, and also to quantify how individually distinctive acoustic features of bellows and size-related information degrade over distance. Our results show that the formant frequencies of bellows derived from Linear Predictive Coding can be used to classify calls to male koalas over distances of 1–50 m. Further analysis revealed that the upper formant frequencies and formant frequency spacing were the most stable acoustic features of male bellows as they propagated through the *Eucalyptus* canopy. Taken together these findings suggest that koalas could recognise known individuals at distances of up to 50 m and indicate that they should attend to variation in the upper formant frequencies and formant frequency spacing when assessing the identity of callers. Furthermore, since the formant frequency spacing is also a cue to male body size in this species and its variation over distance remained very low compared to documented inter-individual variation, we suggest that male koalas would still be reliably classified as small, medium or large by receivers at distances of up to 150 m.

## Introduction

Several studies have shown that the acoustic features of mammal vocal signals encode information about important attributes of the caller, such as its identity, size, sex, reproductive and emotional state, and playback experiments have demonstrated that receivers use this information during sexual or social interactions [1]. What is often neglected in these studies, however, is an evaluation of the distances over which this information is effectively transmitted (but see: [2], [3]–[7]). Indeed, selection should favour vocal signals that effectively advertise specific traits [8]–[13] and resist degradation in a given species’ natural environment [14]–[16] to ensure that this information is efficiently propagated over relevant distances. Moreover, by examining how increasing distance affects the acoustic structure of vocal signals we can gain insights into the potential distances over which information transfer can occur (the ‘active space’ of a vocal signal: [17], [18], [19]). This not only impacts on how animals space themselves in a given environment, particularly in dispersed communities, but also allows us to make inferences about what information animals could be extracting from calls at different distances, giving insights into the function of different vocal signals and the signalling ecology of a species [20].

Attenuation and degradation of vocal signals imposes limits on the distances over which effective communication can occur [14], [21]–[24]. As sound waves travel through the environment they are subject to an approximate 6 dB loss of amplitude per doubling of distance (spherical spreading) and additional ‘excess’ attenuation caused by atmospheric absorption, scattering and deflection by stratified media, and ground attenuation [22], [25], [26]. In addition, atmospheric turbulence and reverberation can limit a receiver’s ability to resolve differences between acoustic features of vocal signals [22], [27]. Furthermore, because attenuation is not consistent across the frequency domain [15], [19], [25], [28]–[31], certain frequency components that are crucial for categorising sounds could be lost or distorted whilst others are not [3], [4], [22], [32]. Consequently, the maximum distance over which a signal can be detected is not necessarily equivalent to the distance over which functionally relevant information can be extracted from calls.

Koalas (*Phascolarctos cinereus*) produce distinctive bellowing calls during the breeding season that are characterised by a series of inhalation and exhalation phases [33]–[35]. Although the precise function of these calls remains unknown, a close correspondence between bellowing and breeding activity [36] indicates that they are likely to have an active role in coordinating this species’ mating activities. Indeed, male koalas often bellow immediately after agonistic interactions and successful or attempted copulations [33], [35] and hence, short-range vocal communication is likely to play a key role during this species’ social interactions. Furthermore, the formant frequencies of male bellows have recently been shown to vary consistently according to the caller’s identity and body size [37], [38]: two attributes of male callers that are potentially important in reproductive contexts. For example, individuals could use bellows to familiarize themselves with calling males during the breeding season, allowing female koalas to exhibit mating preferences based on familiarity and male koalas to avoid known rivals that represent a threat to them. In addition, acoustic cues to male body size could have utility in this species’ sexual communication for assessing rivals and mating partners [39], [40]. This information, however, needs to be propagated reliably in the *Eucalyptus* forest environments that koalas inhabit if it is to be functionally relevant in this species’ vocal communication system.

The primary aim of this study was to determine the distances over which male koala bellows could reliably signal the identity and body size of male callers in this species’ natural environment. To this end, five bellows from each of 10 male koalas (50 in total) were played back and re-recorded over a range of distances in a mixed *Eucalyptus* plantation so that we could: 1) determine the extent that individual classification of male koala bellows becomes less accurate over distance; and 2) quantify how individually distinctive acoustic features and size-related information degrade over distance. Our findings will allow us to estimate the distances over which male koala bellows could reliably signal the identity and size of male callers, and may have important practical implications for noninvasively estimating population levels in this species.

## Materials and Methods

### Ethical Statement

This work follows the Association for the Study of Animal Behaviour/Animal Behaviour Society guidelines for the use of animals in research.

### Original Recordings

The original male bellows used in the transmission experiments were recorded from 10 adult koalas (aged 3–15 years) at Lone Pine Koala Sanctuary, Brisbane, Australia (LPKS), using a Sennheiser ME67 directional microphone and a Zoom H4N portable solid-state digital recorder (sampling rate: 44.1 kHz, amplitude resolution: 16 bits) at distances ranging from 1–5 m. Recordings were transferred to an Apple Macintosh Macbook Pro computer, normalized to 100% peak amplitude and saved as WAV files (44.1 kHz sampling rate and 16 bits amplitude resolution). For the transmission experiments we randomly selected five bellows with very low levels of background noise from each of the 10 male exemplars.

### Experimental Site and Conditions

Experiments were carried out in a mixed *Eucalyptus* plantation at LPKS. The plantation had a density of 1 tree per 40 m^2^ and consisted of *E. mollucana* (gumtop), *E. microcorys* (tallow wood), *E.* *tereticornis* (blue gum), and *E. grandis* (flooded gum). A total of three recording sessions were conducted on the 5^th^, 6^th^ and 8^th^ November 2011, which is during this species acknowledged breeding season. Re-recording experiments were conducted between 0300–0500 when temperature and humidity are lowest and hence, when sound propagation is maximised [22]. The occurrence of male bellowing also remains high at these times in free-ranging koalas [36]. Recordings made on all three recording dates were analysed and the average acoustic values from each call form the basis of the results. For all recording sessions the wind speed was less than 4 kph (measured using a Siemens anemometer) and the temperature and humidity varied between 14–16 degrees Celsius and 87–90%, respectively.

### Experimental Design and Field Recordings

To examine how identity and size-related information degrades with distance from the caller the 50 male bellows were played back and re-recorded over distances of 1 m, 25 m, 50 m, 100 m, and 150 m. Male bellows were broadcast using a Chiayo Focus 505 loudspeaker (Taipei, Taiwan) with a flat (±3 dB) frequency response from 50–15000 Hz. This covered the frequency range of the acoustic measures we considered in our analysis (see table 1). Bellows were played back 2 s apart, and at mean sound pressure levels of 75 dB measured 1 m from the source (determined using a Radio Shack Sound Level Meter, set for C-weighted fast response). Previous sound pressure level measurements taken approximately 1 m from 10 different vocalising male koalas indicates that this is equivalent to that of naturally bellowing males (*N* = 10, mean = 73 dB). Bellows were re-recorded using a Zoom H4N portable solid-state digital recorder (sampling rate: 44.1 kHz, amplitude resolution: 16 bits) and a RODE NTG-2 directional microphone fitted with a RODE blimp (windshield).

**Table 1:** Descriptive statistics for the acoustic measures of the re-recorded bellows (*N* = 50).

Acoustic measures	Mean	s.d.	Minimum	Maximum
F1	245.7	21.5	212.0	318.5
F2	484.5	32.9	409.0	564.0
F3	734.2	71.7	592.5	917.5
F4	1202.8	71.0	1066.5	1310.5
F5	1663.5	85.7	1490.0	1826.5
F6	2156.1	109.1	1961.5	2361.0
ΔF	367.0	14.1	344.8	399.5

Koalas typically remain at the level of the tree canopy in their natural environments in order to eat and obtain shelter (B. D. Charlton & W. A. H. Ellis, Pers. Obs.). Consequently, the loudspeaker and microphone were fixed on tripods at a height of 4 m above the ground, which corresponded to the canopy height in the *Eucalyptus* plantation. This propagation height also represents a typical male koala signaller and receiver position in natural conditions (B. D. Charlton & W. A. H. Ellis, Pers. Obs.). The speaker and microphone were both free standing and not within 2 m of any foliage. For each re-recording distance the speaker remained in a fixed location while the microphone was moved, and the speaker and microphone were always oriented towards each other. Re-recording distances were measured and marked out during the hours of daylight using a Bushnell Yardage Pro laser rangefinder.

### Acoustic Analyses

**Figure 1:**
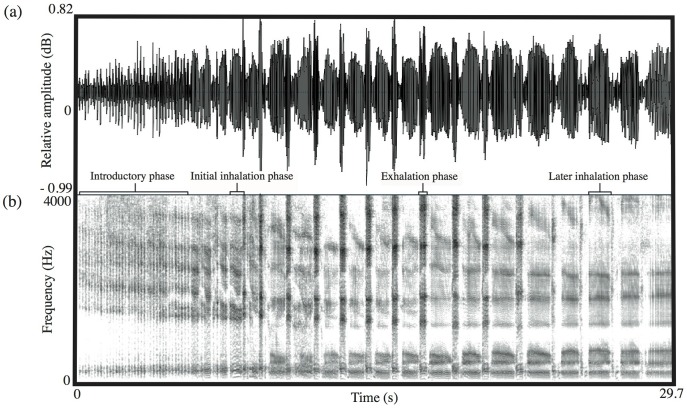
Waveform (a) and spectrogram (b) of a male bellow. Spectrogram settings: FFT method; window length 0.05 s; time step = 0.004 s; frequency step = 10 Hz; Gaussian window shape; dynamic range = 35 dB. Male bellows are characterised by an introductory phase that is followed by a continuous series of inhalations and shorter exhalations.

All acoustic analyses were conducted using Praat 5.0.29 DSP package (www.praat.org). Koala bellows consist of a continuous series of inhalations and shorter exhalations, and often have an introductory phase [35] (see figure 1). Previous work has shown that the most reliable cues to identity and body size in male bellows are the frequency values of the first six formants of the inhalation phases and the overall formant spacing derived from these frequency values [37], [38]. We did not attempt to measure formants higher than the sixth formant because these frequency components are often poorly defined and their information content is not known [37], [38]. In addition, other acoustic features, such as bellow fundamental frequency (pitch), duration, mean and maximum amplitude, are not reliable cues to identity or size in this species [37], [38] and thus, were not considered in the analysis. For the acoustic analysis we selected two inhalation phases with low background noise from each of the 50 male bellows. The same inhalation sections re-recorded at different distances were then extracted and saved as separate WAV files using the labelling facility in Praat.

The mean frequency values of the first six formants of the inhalation phases were measured using Linear Predictive Coding (LPC; ‘To Formants (Burg)’ command in Praat) and the following analysis parameters: time step: 0.01 seconds; window analysis: 0.2 seconds; maximum formant value: 2500 Hz; maximum number of formants: 6; pre-emphasis: 50 Hz. In order to more accurately measure the lower three formants we ran a second analysis in which the maximum formant value was changed to 1000 Hz and the maximum number of formants to three. The formant values from both analyses were then combined (F4, F5, F6 from the first analysis and F1, F2, F3 from the second analysis) and the average formant frequency values were used to estimate the formant spacing (ΔF) [12] achieved during each vocalisation. To do this, the measured formant values were regressed against those that would be expected in a straight uniform tube closed at one end (the glottis) and open at the other end (the mouth) [41].

### Individual Classification of Male Koala Bellows Over Distance

A Discriminant Function Analysis (DFA) was used to classify bellows at the different re-recording distances with subject identity entered as the group identifier and the mean values of the acoustic measures (F1, F2, F3, F4, F5, F6 and ΔF) as discriminant variables. Although some of our acoustic measures were correlated, we wanted to retain the raw variables in the analysis to minimize loss of information [37]. To assess the stability of vocal individuality over distance we took four bellows from each of the 10 male exemplars re-recorded at 1 m, and used them as a training set for models to classify observation bellows re-recorded at 25 m, 50 m, 100 m, and 150 m to each of the 10 male exemplars (‘hold-out-sample’ method: [42], [43]). Thus, the training set comprised 40 bellows re-recorded at 1 m, which was then used by the DFA to classify the remaining 10 bellows to each individual over distance (hence, different bellows were used for training and classification). Koalas often bellow immediately after agonistic interactions and successful or attempted copulations [33], [35]. We have assumed, therefore, that vocal signals are linked to individuals and particular behavioural outcomes at ranges of <25 m and hence, that koalas attend to relatively un-degraded bellows when they do this. The DFA was conducted using IBM SPSS statistics version 19. The statistical significance of correct classification was obtained using the Chi square statistic, significance levels were set at 0.05, and two-tailed probability values used.

**Figure 2:**
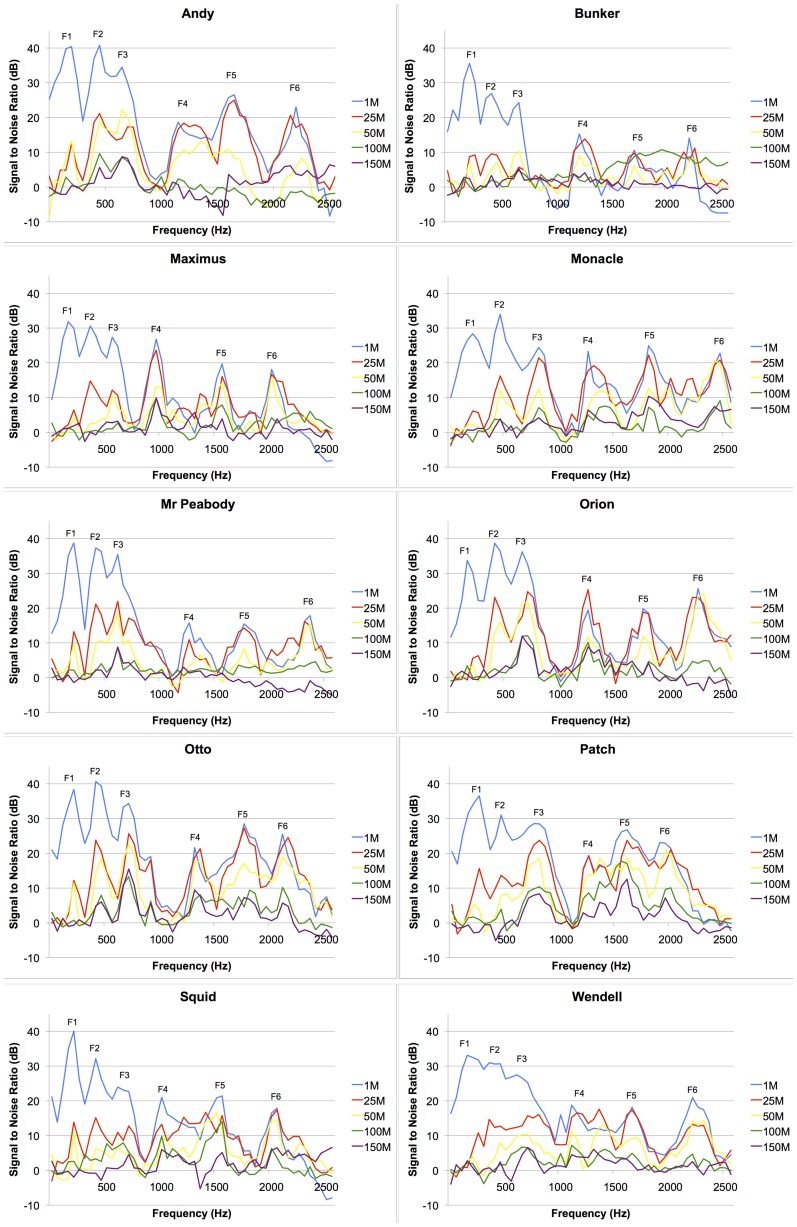
SNR plots. The plots illustrate how the frequency amplitudes of bellow inhalation sections from 10 male koalas attenuate over distance. Missing values indicate that the call was not detectable at the re-recording distance.

### Analysis of Call Degradation

We decided to quantify the degradation of male bellows in two ways: by examining how frequency components deviate from their original values as they propagate (stability); and by assessing how the relative amplitudes of frequency components drop over distance (amplitude attenuation).

#### Stability of individual acoustic measures

To examine the stability of individual acoustic measures over distance we used relatively un-degraded bellows re-recorded at 1 m as reference bellows, and calculated the absolute percentage (%) difference between the formant frequencies (F1–F6) and ΔF of reference bellows and bellows at each subsequent re-recording distance. In addition, we compared the averaged acoustic parameters for each of the 10 male exemplars at each rerecording distance to those obtained from relatively un-degraded bellows rerecorded at 1 m (reference bellows) using the Pearson product-moment correlation [5], [6]. Average acoustic measures were calculated using the 5 bellows from each male. We considered positive correlations >0.5 as indicating that acoustic features were stable across distances and hence, that receivers would be able to reliably use these features to assess the identity and size of male callers.

#### Amplitude attenuation

Re-recordings from each of the 10 male exemplars were also used to assess how the formant frequency amplitudes of male bellow inhalation sections degrade with distance from the caller. To do this a long-term average spectrum (LTAS) of each inhalation section was used to quantify the amplitude attenuation across frequencies averaged over the duration of these sections at each distance [32]. LTAS were generated using a frequency analysis window of 50 Hz, yielding 52 quantitative variables (H1–H52) that show the amplitude (dB) of 50-Hz frequency slices (H1∶0–50 Hz; H2∶50–100 Hz to H52∶2550–2600 Hz). Before comparing levels of amplitude degradation over different distances, however, it is important to determine the variations in the background noise and its likely contribution to the overall variation.

Consequently, the LTAS of a 1 s segment of background noise immediately following an observation bellow was used to estimate frequency distribution of energy in the noise component. The LTAS of this noise segment was then subtracted from the LTAS of the bellow inhalation section to determine the ratio of the level of the call plus background noise to that of background noise alone, termed the Signal to Noise Ratio (SNR) (see figure 2). To objectively assess the amplitude degradation of individual formants the peak SNR of F1–F6 at 1 m, 25 m, 50 m, 100 m and 150 m for each of the 10 male exemplars were regressed with re-recording distance, allowing us to generate regression slope values to quantify how formant amplitudes dropped over distance.

## Results

### Individual Classification of Male Koala Bellows Over Distance

Using DFA’s trained with bellows re-recorded at 1 m resulted in reclassification levels of 50% for bellows rerecorded at 25 m, 30% for those rerecorded at 50 m, 20% for those rerecorded at 100 m, and 10% for those rerecorded at 150 m (see table 2). The level of re-classification to caller identity achieved was statistically significant at 25 m and 50 m (see table 2). In contrast, re-classification of bellows re-recorded at 100 m and 150 m did not approach statistical significance (see table 2). These results suggest that koalas are unlikely to use male bellows to recognise individuals at distances greater than 50 m. The main contributors to individual vocal distinctiveness were the upper formants (F3–F6) and ΔF (see table 3).

**Table 2:** Observed % of correct reclassification against expected levels.

Distance (m)	Expected	Observed	*X^2^*	*p*
25	10%	50%	160	<0.001
50	10%	30%	40	<0.001
100	10%	20%	10	>0.1
150	10%	10%	0	>0.1

The DFA was trained to classify observation bellows re-recorded at 25 m, 50 m, 100 m, and 150 m to each of the 10 males using bellows re-recorded at 1 m. The statistical significance of correct classification was obtained using the Chi square statistic.

**Table 3:** DFA structure matrix.

Acoustic measures	Discriminant functions			
	1	2	3	4
F1				
F2			0.53	
F3		0.48	0.44	−0.72
F4	0.41			0.47
F5	0.68			
F6		−0.71	0.76	
ΔF	0.75			
% of variance	62.6	23.4	7.8	4.8

The table shows pooled within-groups correlations among discriminating variables and the four standardized canonical discriminant function with eigenvalues >1. Correlations >0.4 are shown.

**Table 4:** Absolute % difference ± s.e. at each re-recording distance.

Acoustic measures	Mean ± s.e absolute % variation at each distance				Mean
	25 m	50 m	100 m	150 m	
F1	14.1±3.0	5.0±1.3	10.2±2.0	16.4±2.2	11.4
F2	6.6±1.1	11.8±1.6	15.6±2.7	10.8±1.7	11.2
F3	4.7±0.8	2.8±1.0	5.0±1.8	5.0±1.7	4.4
F4	1.1±0.2	1.3±0.3	2.3±0.5	3.5±0.6	2.1
F5	3.5±0.4	7.1±1.0	7.2±1.3	6.6±1.2	6.1
F6	1.1±0.2	2.5±0.4	4.9±0.9	4.7±2.6	3.3
ΔF	0.9±0.2	1.8±0.4	2.9±0.7	3.0±0.7	2.2

Acoustic measures at 1 m (reference calls) were compared with those re-recorded at the other distances.

### Analysis of Call Degradation

#### Stability of individual acoustic measures

In general, the absolute % variation and correlation coefficients for all the acoustic measures decreased over distance, indicating that the stability of individual vocal characteristics decreased as the distance between the speaker (signaller) and microphone (receiver) increased (see tables 4 and 5). F4 and ΔF were the most stable acoustic measures, having the lowest absolute % variation and highest correlations between their values in reference bellows and those re-recorded at 150 m (see tables 4 and 5). Since ΔF is a cue to male koala body size [38], these findings indicate that size-related information is relatively stable over distances of 1–150 m. In contrast, F1 and F2 yielded the highest absolute % variation and lowest correlation coefficients and, therefore, the poorest stability (see tables 4 and 5).

**Table 5:** Pearson product-moment correlation coefficients.

Distance (m)	F1	F2	F3	F4	F5	F6	ΔF
1–25	−0.13	**0.71**	**0.96**	**0.98**	**0.96**	**0.99**	**0.99**
1–50	0.22	0.33	**0.92**	**0.93**	**0.86**	**0.98**	**0.97**
1–100	−0.28	−0.07	**0.82**	**0.89**	**0.64**	**0.95**	**0.82**
1–150	−0.25	0.12	**0.69**	**0.93**	**0.69**	**0.72**	**0.82**

Comparisons were made between acoustic measures at 1 m (reference calls) and those re-recorded at the other distances (*N* = 10). Values in bold denote correlations >0.5.

#### Amplitude attenuation

**Figure 3:**
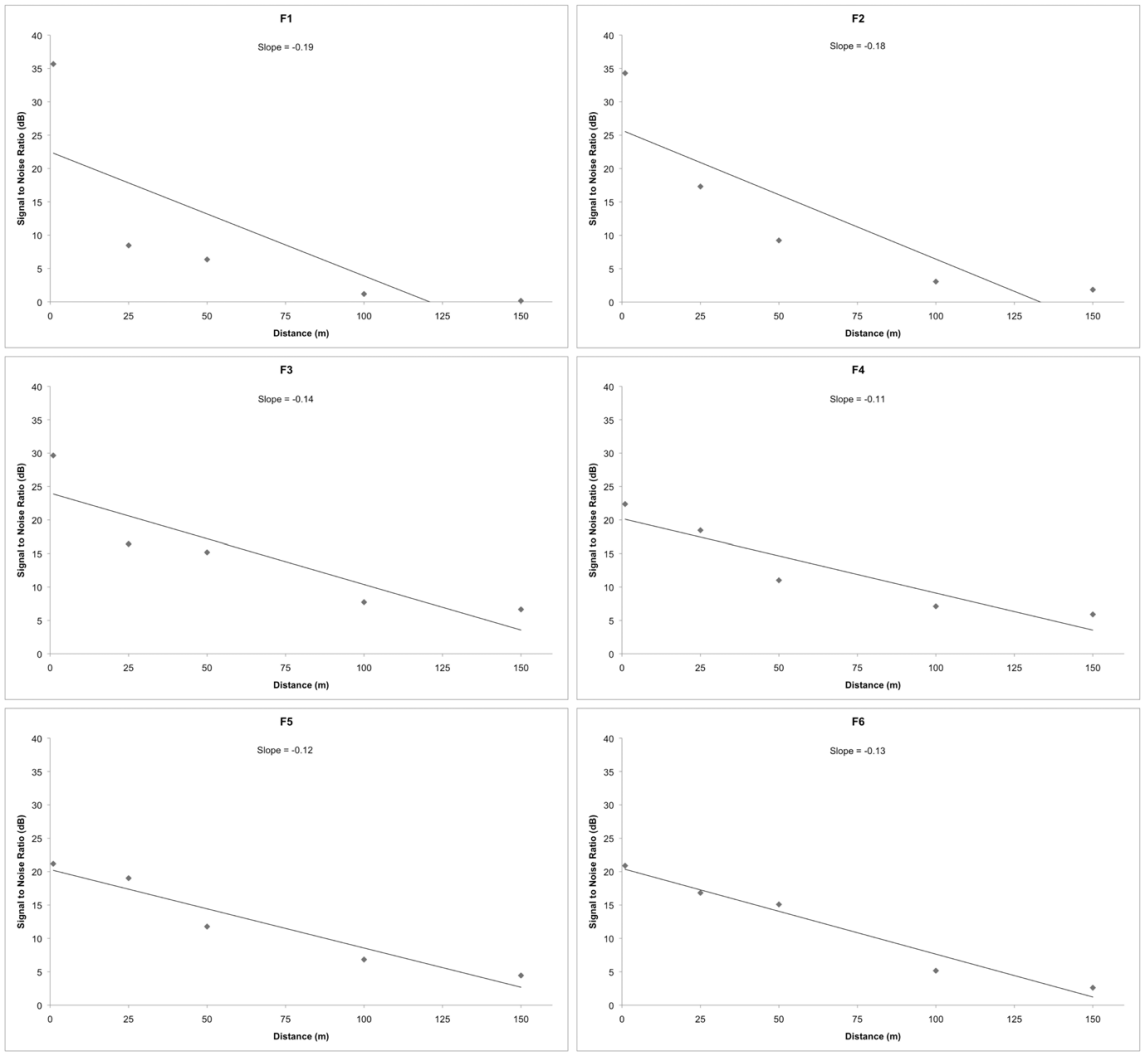
Mean SNR values and regression lines for individual formants at the different distances. The regression slope values quantify how the formant amplitudes of re-recorded bellows drop over distance (*N* = 10). Higher regression slope values indicate greater amplitude attenuation over distance.

Figure 2 depicts how the inhalation sections of a single bellow from each of the 10 male exemplars degrade over distance. Visual assessment of the SNR plots generated by LTAS subtraction indicates that the lower formants F1 and F2 had the highest regression slope values and thus, were the most susceptible to decay as they propagate through the *Eucalyptus* canopy (see figure 3).

## Discussion

The results of the current study show that individual vocal distinctiveness in male koala bellows remains stable over distances of up to 50 m. These findings suggest that conspecific receivers could recognise different male koalas using their bellow vocalisations over these distances. Previous work has shown that male and female koalas can discriminate between male callers on the basis of the bellow vocalisations [37]; however, since acoustic features could become distorted as they propagate through the environment, the ability to vocally recognise different male koalas may become compromised. The present study confirms that vocal recognition of male koalas is unlikely to occur at distances greater than 50 m, and indicates that the main contributors to individual vocal distinctiveness are the upper formants (F3–F6) and ΔF. Although our results suggest that koalas are unlikely to *recognize* known individuals at distances greater than 50 m, we cannot rule out the possibility that koalas progressively learn to do so over greater distances, placing more importance on individually distinctively acoustic features (like F4 and ΔF) that are relatively stable as they propagate through the environment.

Furthermore, vocal recognition and the ability to signal individual identity may still be important in this species’ inter and intra-sexual communication when individuals come into close proximity. At this time, the ability to distinguish between unfamiliar and familiar rivals could help prevent unnecessary contests between males [44], and over the course of a breeding season, females may become familiar with and prefer the vocalisations of certain males that can afford higher energy courtship displays [45]–[47]. Based on the findings of the current study and the fact that the home range size of a male koala during the breeding season is around 6.6 ha [48], however, it seems unlikely that male koalas could use bellows for delineating territories [49], [50].

It is also worth noting that different environmental conditions and propagation heights will alter the distances over which this type of information could be reliably transmitted. For example, lower ambient temperature, humidity and wind noise will all contribute to greater sound propagation distances [22], and these variables could also be affected by the caller’s elevation. Our study was conducted using vocalizations re-recorded with almost no wind noise and at a time just before dawn when temperature and humidity are lowest (0300–0500). In addition, our chosen propagation height of 4 m corresponds well with typical male koala signaller and receiver positions in natural conditions (B. D. Charlton & W. A. H. Ellis, Pers. Obs.). Consequently, our results reflect propagation from a species-typical calling position in optimal conditions [22], [26].

Our analysis of call degradation indicates that F1 and F2 were the least stable features of male bellows, and also that these formants were more severely attenuated than the others as they propagate through the *Eucalyptus* forest environment. These results are surprising given that lower frequencies are predicted to propagate best in forest environments [15], [19], [25], [28]–[31]. Excess attenuation of lower frequencies due to ground reflections is unlikely to provide an explanation because we broadcast and re-recorded male bellows at a height of 4 m [25], [26]. One possible reason that F1 and F2 are absorbed at higher rates than the other formants as they propagate, however, is frequency dependant reverberation [22].

Reverberation is caused by reflection or scattering of sound waves that later re-join the main beam of sound propagation [26] and, because of reflections from tree trunks and foliage, it is considerably greater in forests than in open areas [22]. Furthermore, previous studies measuring habitat-specific attenuation of artificial signals in forest environments imply that reverberation over distance is more pronounced for frequencies below 1 kHz than it is for frequencies between 1 and 3 kHz [27], [51]. Accordingly, since formants F1 and F2 of male bellows fall below 1 kHz (at around 200 Hz and 400 Hz, respectively: see figures 1 and 2) this observation may explain why they are more severely attenuated and less stable than the upper formants that fall within the 1–3 kHz range. In addition, because other nonhuman mammals can perceive formant shifts of 8–10% [52], [53] most of the variation in F1 and F2 over distance is likely to be perceptible to koalas. In contrast, most of the variation in other acoustic measures (of between 0.9 and 7.2%) would probably not be salient (as in humans: [54], [55]). Taken together then, these findings indicate that koalas should attend to variation in the upper formants and ΔF when assessing the identity of callers.

Indeed, ΔF was a highly stable acoustic feature of male koala bellows, remaining strongly correlated over distance with its value in reference bellows rerecorded at 1 m, and varying over distance by a maximum of 3% at 150 m (see tables 4 and 5). Since this variation is known to be below the just noticeable difference for humans it may not be perceptible to koalas [54], [55]. Furthermore, a 3% variation in ΔF remains small compared to the inter-individual variation reported for male koalas of 31% [38] and, therefore, is not likely to be functionally meaningful when assessing the body size of male callers i.e. individuals would still be correctly classified as small, medium or large by receivers. As a result, ΔF would also appear to be a reliable acoustic cue to body size over distances of 1–150 m in this species’ typical environment and thus, size-related formant information could be reliably broadcast over this range.

Male koalas have a home range size of around 6.6 hectares during the breeding season [48]. If we assume that ranges are roughly circular, then a male positioned in the centre of his range would be around 145 m from the outer boundary (radius = √(area/π)). Thus, our findings indicate that male koalas could effectively propagate size-related information over their home range. Moreover, the effective signalling of size cues over distances of 150 m would allow males to make appropriate decisions about whether or not to enter the home ranges of potential rivals, and may help to explain the observed home range sizes of male koalas during the breeding season.

Our findings also have implications for bio-acoustic population monitoring. Assessing koala population sizes can be difficult because koalas are relatively cryptic and typically occur in low abundance [34]. However, since male koala bellows are individually distinctive and delivered at high rates during the breeding season [35], [36], bio-acoustic monitoring of population sizes in this species is a realistic possibility. On the basis of our results, we suggest that recording devices are placed a maximum of 50 m apart in a given environment, so they can reliably capture enough acoustic information to classify bellows to different individuals over distance (i.e. recognise different individuals and not over inflate population estimates by counting individuals more than once). In addition, bellows should be classified to individuals using the most reliable individually distinctive features. The results of this study accord with previous work [37] in showing that the upper formants and ΔF are the most individually distinctive features of male koala bellows, and also reveal that these features of male bellows propagate best in this species’ *Eucalyptus* forest environment. Accordingly, we suggest that bio-acoustic population monitoring in this species should focus on measuring the upper formants of male bellows, in order to accurately classify vocalisations to individuals.

In sum, we have shown that individual vocal distinctiveness in male koala bellows remains stable over distances of up to 50 m in this species’ typical environment. In addition, the upper formants of male koala bellows appear to propagate best through the environment, possibly utilising a 1–3 kHz window of minimum frequency dependant reverberation in forest environments [27], [51]. Moreover, while the upper formants of male bellows might be sufficient to discriminate between individuals [37], the overall pattern of formants may be important for vocal recognition. This could constrain the vocal recognition process to distances of less than 50 m in the *Eucalyptus* forest environments that koalas inhabit, due to distortion of the lower formants, and has implications for bio-acoustic population monitoring in this species. In contrast, ΔF was the most stable acoustic feature of male bellows, varying by a maximum of 3% as it propagates, and suggesting that size-related information is reliably broadcast at distances of up to 150 m in this species’ typical environment.

Finally, these findings permit us to make a first approximation of the active space [17]–[19] of koala vocalizations. By limiting the distances over which effective communication can occur this parameter provides important insights into the signalling ecology of a species. For instance, vocal recognition and the ability to signal individual identity may only be important when individuals are less than 50 m apart. Within this range, the ability to distinguish between unfamiliar and familiar rivals could help prevent unnecessary contests between males [44], and females may become familiar with and prefer the vocalisations of certain males that can maintain close proximity to them as they approach and enter oestrous [56]. In addition, koalas could use ΔF to reliably assess the body size of male callers in reproductive contexts at distances of up to 150 m, and make appropriate decisions about whether or not to interact with potential mates and rivals based on the size-related information in bellows [39], [40], [57]. Future studies should use re-synthesis techniques to shift individual formants and a habituation–discrimination playback paradigm [52] to investigate the importance of individual formants for vocal recognition in this species. In addition, the ability of koalas to *recognise* specific individuals could be examined by presenting the vocalisations of the same familiar individual separated by a few seconds from two different places located several meters apart (a physically impossible scenario) versus a realistic scenario in which the vocalisations of two different familiar individuals are played back [58]. Playback experiments could also quantify the response of free-ranging koalas to formant-shifted bellows simulating different size males broadcast at different distances from target animals. This approach will allow us to ascertain whether true vocal recognition occurs in koalas, and also to determine the active space for identity and size cueing in this species’ natural settings.
